# What are the constraints and opportunities for HIVST scale-up in Africa? Evidence from Kenya, Malawi and South Africa

**DOI:** 10.7448/IAS.18.1.19445

**Published:** 2015-03-20

**Authors:** Heidi van Rooyen, Olivia Tulloch, Wanjiru Mukoma, Tawanda Makusha, Lignet Chepuka, Lucia C Knight, Roger B Peck, Jeanette M Lim, Nelly Muturi, Ellen Chirwa, Miriam Taegtmeyer

**Affiliations:** 1 Human Sciences Research Council, Durban, South Africa; 2 Liverpool School of Tropical Medicine, Liverpool, United Kingdom; 3 Liverpool VCT, Care, and Treatment, Nairobi, Kenya; 4 Kazumu College of Nursing, Blantyre, Malawi;; 5 School of Public Health, University of Western Cape, Cape Town, South Africa;; 6 PATH Seattle, WA, USA

**Keywords:** HIVST, country readiness, policy and political support for self-testing, HIV testing, opportunities and limitations of self-tests

## Abstract

**Introduction:**

HIV self-testing (HIVST) has the potential to increase uptake of HIV testing among untested populations in sub-Saharan Africa and is on the brink of scale-up. However, it is unclear to what extent HIVST would be supported by stakeholders, what policy frameworks are in place and how variations between contexts might influence country-preparedness for scale-up. This qualitative study assessed the perceptions of HIVST among stakeholders in three sub-Saharan countries.

**Methods:**

Fifty-four key informant interviews were conducted in Kenya (*n*=16), Malawi (*n*=26) and South Africa (*n*=12) with government policy makers, academics, activists, donors, procurement specialists, laboratory practitioners and health providers. A thematic analysis was conducted in each country and a common coding framework allowed for inter-country analysis to identify common and divergent themes across contexts.

**Results:**

Respondents welcomed the idea of an accurate, easy-to-use, rapid HIV self-test which could increase testing across all populations. High-risk groups, such as men, Men who have sex with men (MSM), couples and young people in particular, could be targeted through a range of health facility and community-based distribution points. HIVST is already endorsed in Kenya, and political support for scale-up exists in South Africa and Malawi. However, several caveats remain. Further research, policy and ensuing guidelines should consider how to regulate, market and distribute HIVST, ensure quality assurance of tests and human rights, and critically, link testing to appropriate support and treatment services. Low literacy levels in some target groups would also need context-specific consideration before scale up. World Health Organization (WHO) policy and regulatory frameworks are needed to guide the process in those areas which are new or specific to self-testing.

**Conclusions:**

Stakeholders in three HIV endemic sub-Saharan countries felt that HIVST will be an important complement to existing community and facility-based testing approaches if accompanied by the same essential components of any HIV testing service, including access to accurate information and linkages to care. While there is an increasingly positive global policy environment regarding HIVST, several implementation and social challenges limit scale-up. There is a need for further research to provide contextual and operational evidence that addresses concerns and contributes to normative WHO guidance.

## Introduction

The global policy framework on HIVST has moved rapidly since the First International Symposium on HIV Self-testing (HIVST) in 2013 debated the legal, ethical gender and human rights aspects and set out a research agenda [1]. This was followed by a review of research and policy priorities [2] and the release of a technical update on HIVST [3] that provided an outline to the steps countries need to take before embarking on HIVST scale-up. The document further outlines the WHO commitment to the development of normative guidance on HIVST once high-quality evidence is available and a better understanding of the challenges has been documented. A recent editorial in a special issue on HIVST summarizes progress and remaining research gaps in realizing the potential of HIVST in the global HIV testing and counselling (HTC) response [4].

As highlighted in a recent paper on the regulatory and policy considerations for HIVST, national HTC policy lags behind this emerging global debate [5]. National HTC strategies usually endorse a basket of approaches to reach target populations and combine provider-initiated testing in health facilities with traditional stand-alone voluntary counselling and testing sites and a range of community outreach approaches. Through this they hope to provide testing to those with symptoms, healthy individuals, couples and families from all sectors of the population. These have had some success but are unable to ensure that countries meet targets of universal testing. Facility-based HTC is constrained by a perceived lack of privacy, long waiting times in facilities, social stigma and the inconvenience and personal costs involved in accessing testing services [6–8]. Community-based approaches such as home and mobile HTC are designed to address these constraints [9–12]. However, critical gaps remain with up to 50% of people untested; key populations, men and adolescents underserved [13,14] and traditional HTC approaches failing to achieve rates of linkage to care and treatment services adequate to ensure public health gains without additional strategies and resources [15–19].

HIVST has the potential to address these gaps by increasing access to confidential testing at the community level in certain groups, providing earlier diagnosis, linkage to care and de-stigmatizing HIV [20–22]. Arguments against self-testing include potential inaccuracy of tests, psychological risks and uncertainty over unsupervised linkage to care [4,23,24]. Delivery and policy concerns have been expressed in introducing self-testing in countries where health infrastructure may be limited.

We present findings from the first part of a two-phase study. The second phase evaluated the usability of HIV self-test prototypes by lay users and is published elsewhere [25]. In the first phase, we undertook a qualitative study to examine the constraints and opportunities for HIVST policy and readiness in three endemic sub-Saharan African contexts: Kenya, Malawi and South Africa. Three countries where HIVST is legal but where policy and guidance have not yet been put in place for scale-up [26]. Kenya has an HIV prevalence of 5.6%; testing coverage of 72% was achieved in 2012 [27]. Recent pilot studies with health workers have been conducted [28] and self-testing is allowed, but not detailed, in HTC policy. Test kits must be approved for use in Kenya and confirmatory testing is required. Operational guidelines and a testing algorithm are under development. In Malawi, the adult HIV prevalence rate is estimated at 12% with 21% of adults undergoing HTC [20]. The National HIV strategy framework and policy does not include HIVST, but pilot studies on HIVST have been published and are highly cited internationally [20,21]. South Africa has an adult HIV prevalence of 12.2% with 44.8% of adults reported being tested in 2012 [29]. HIV policy does not currently cover HIVST, there is no mechanism in place to regulate the quality and reliability of self-tests [30] and very few empirical studies on the topic have been published, although pilots with health care workers have been conducted [31]. We used a qualitative approach to gain in-depth insight to the issues relating to HIVST in the three countries and capture the diversity of opinions, experiences and attitudes [32].

## Methods

Data were collected between June and August 2013. We conducted key informant interviews (KII) with stakeholders who were purposively sampled in order to achieve a range of different perspectives of those working in HIV programming, including those with decision-making authority regarding HIV testing strategy and policy; HIV test procurement, and researchers. Once participants were briefed on the study and indicated willingness to participate, written informed consent was obtained.

It was not always feasible to interview respondents in all categories (see Table 1). Our approach was both deductive and inductive. First a common research framework was established and themes for generic interview topic guides were developed collaboratively. Questions included: the level of interest in HIVST both personally and in the country; groups most likely to use an HIVST kit; appropriate distribution channels; regulatory requirements, distribution of self-test kits, main target populations, test kit features (with probes on specific characteristics, performance and usability), how to achieve linkage to care, benefits and challenges of self-tests and strategies for scale-up, cost of test kits, consent procedures in HIVST (the generic topic guide is found in Supplementary file A). Adaptations were made to topic guides by the country researchers to allow for contextual differences and further changes were made after piloting topic guides, for example to include questions about country readiness and political will. Interviews took 45 minutes to 1 hour to conduct.

**Table 1:** Key informant interview respondents (number approached in brackets)

Respondent type	Kenya	Malawi	South Africa
Government	3 (3)	10 (10)	2 (3)
NGO	4 (5)	9 (9)	4 (5)
Academic/research	3 (3)	3 (3)	3 (3)
Procurement agency	2 (3)	0 (1)	1 (1)
Donors	0 (3)	3 (3)	2 (2)
Lab	4 (4)	1 (1)	0 (2)
Total	16 (21)	26 (27)	12 (16)

### Analysis

Interviews were primarily conducted in English, recorded, transcribed verbatim and imported into NVivo v9 software for management and analysis. In Malawi, some interviews were done in Chichewa and translated to English for analysis. Each country developed a coding framework. These were shared, refined and agreed between partners to allow for inter-country analysis so that each country coded data to the same major themes. Data were coded independently in each country starting with common sub-themes and adding context-specific themes as appropriate before and during the coding process. Analysis and findings were discussed on a regular basis by teleconference and separate country reports written. The NVivo country project files were harmonized by an independent researcher who undertook the final thematic inter-country analysis referring to these and the raw data, this additional layer of analysis enabled checking for inter-coder reliability and ensured further trustworthiness of the analysis process. Researchers from each partner gave input and comments to the inter-country analyses.

### Ethics

Ethical approval was obtained from the Kenya Medical Research Institute (KEMRI) Ethics Review Committee, the Malawi College of Medicine Ethics Committee (COMREC) and the Research Ethics Committee of the Human Sciences Research Council (HSRC) in South Africa.

## Results

We interviewed 54 key informants with an overall response rate of 84.4%. The results are structured on three levels: perceptions on policy; perceptions on implementation; and concerns, challenges and perceived evidence gaps. They include responses to both opportunities and constraints on HIVST scale-up. The views on constraints or opportunities of HIVST described here were initiated by respondents unless otherwise stated (refer also to Supplementary file A topic guides). Illustrative quotations from key informants are shown in the main body of text and in boxes and are attributed with the country and sector in which the stakeholder worked.

### Policy: enthusiasm, readiness and regulation

In all three countries key informant respondents would welcome the introduction of a rapid HIV self-test. However, when questioned on the readiness for self-testing in their contexts they cited a need for caution, identifying areas that would require action at national level before the test could be rolled out.

Setting up a regulatory and policy framework in line with WHO guidance and adapted to local conditions was considered essential by most informants. As shown in Table 2, some respondents in South Africa and Kenya felt these countries were ready to develop a self-testing policy, but clear regulatory guidelines which covered the features of self-testing devices as well as the broader self-testing programme needed to be set up first. South African respondents were most vocal about regulatory issues, and although the need for legal and regulatory frameworks were noted by Kenyan and Malawian respondents, the interviewers probed less on this issue and there was much less volunteered about this issue in Malawi in particular.

**Table 2:** Quotations discussing regulatory needs

Kenya	“Self-testing is something that we have heard is going on in an informal manner so if that is happening we need policy makers to try and introduce a structured way of this happening … we need to come in and streamline” (Government)
Malawi	“I think it's a process for the Ministry to harness, because as of now we have not formally taken HIV self-testing as a national policy knowing the challenges that go with it. You know, HIV testing itself requires a lot of modification at the moment” (Government)
South Africa	“In America the FDA very clearly regulates medical devices, Europe has a very clear regulatory system for medical devices, the problem comes in a country which does not have a good regulatory system” (Government) “… the legal framework might look at and think of infection controls, think of again domestic violence, think of the famous one – people would commit suicide if they perform the test and they are not counselled … I think that our legal framework should be looking at these and seriously considering making room for self-administered HIV test” (Donor)

Key concerns for all settings included: how surveillance, usage and effectiveness data will be captured to assess the impact of self-testing; ensuring quality assurance of test kits; safeguarding reliable procurement and distribution of test kits; improving test accuracy; developing testing algorithms and protocols for confirmatory testing and ensuring linkage to care.

Country-specific differences about other policy and regulatory matters included marketing self-tests; test disposal; dealing with human rights abuses such as coercion or domestic violence (South Africa and Kenya); controls against counterfeit tests, laboratory regulations (Kenya); legislation about disclosure, (South Africa) and regulation of logistics and supplies (Malawi).

While there were several limitations to the current HIVST system in all three countries, respondents felt the potential benefits of self-testing in identifying new infections that could benefit from existing care and treatment services outweighed the risks of the model. Some respondents expressed an eagerness not to delay the introduction of self-testing, providing adequate consultation with stakeholders occurs first (see Table 3).

**Table 3:** Quotations illustrating level of enthusiasm and readiness for scale-up

Kenya	“you have to think very carefully how … before you even scale up, what are you scaling up? Is it a service? Is it a kit? Is it an opportunity to make decisions, and who is the target population?” (Academic) “We need to look at all avenues so that people can access testing services at the moment … we think that the introduction of self-testing in this country is going to actually help us to achieve that target (80%) … The more we test the more we are able to identify and the more we are able to link to care and treatment. So that is what we are looking for.” (Government)
Malawi	“In my own thinking I would probably think that the country may not be ready, but it does not mean that we cannot do anything about it” (Government) “It has to be ready, we have no choice we have to be ready (for self-testing scale-up) … because infrastructure is in place and people are there, they can be oriented and the public can be motivated to say ‘ok I also need to do that, I don't want to go to the hospital for a test … let me do it alone then once I find out I can start going to another level of care’” (Government) “I think the levels of acceptance are still low that's why we still have stigma and discrimination in relation to people living with HIV. It becomes a big issue if we just say, ‘today everyone can just test himself or herself’ … (Malawi) is not yet ready but we can prepare it to get ready.” (Government)
South Africa	“I think so! The government's response at least on a political level is at least well informed and is open to what proves to work. There is a very sound and realistic sense of what should be done to address the issue of HIV. I think there is government will, I don't know if there is money but on a political level I guess they will be close to introducing this … ” (NGO) “Every South African should be tested at least once a year … we need to have various approaches of reaching people, innovative and yet user friendly” (Government)

### Implementation: target population, distribution and costs

#### Target population

Respondents from all three countries felt that one of the most important benefits of self-testing was the potential to encourage testing in previously untested groups. In particular, HIVST could be desirable for users concerned with stigma or confidentiality associated with existing HCT. Men, couples, MSM[MSM], sex workers and adolescents were considered key populations who could benefit from HIVST. Table 4 shows the range of potential targets suggested by key informants.

**Table 4:** Target populations

Target	Kenya	Malawi	South Africa
Men	√√	√√	√√
Sex workers	√√	√	√
Youth/adolescents	√√	√√	√√
MSM	√√	√	√√
Any/everyone	√	√√	√
Couples	√√	√√	√√
MARPs: including sex workers, truck drivers,^a^ IDUs	√√		√√ [IDUs]
Young and pregnant women	√	√	√
Fishing and other migrant communities		√	
Slum dwellers	√		√
Those worried about confidentiality at facilities	√		
Rural people		√	√
Repeat testers		√	√
Middle class working people [office workers]	√	√	√
Middle aged people		√	

a This is not a quantitative assessment;

however √= suggested and √√= frequently suggested.

Several additional country-specific nuances regarding target population for HIVST emerged. In 2012, the Kenyan government had an 80% coverage target for HIV testing [33]. Progress towards this goal has plateaued and stakeholders felt that HIVST could re-ignite the national effort. In South Africa, there was a strong sense that self-testing would appeal not only to those who choose to avoid existing services, but also for high-risk groups who require more frequent testing.

#### Distribution

The appropriate channels for distributing self-tests can be divided into two main types: existing facilities and sites, or, distribution through alternative settings not normally associated with health services. As depicted in Table 5, the choice of distribution model largely appeared to depend on the target population and many distribution channels were suggested.

**Table 5:** Quotations on suggested distribution channels

Kenya	Pharmacies, mobile outreach at HIV hotspots, workplace (with mass counselling), public sector facilities, General Practitioners, lay counsellors, kiosks
Malawi	Health facilities, CBOs, NGOs, HSAs, existing support groups, peer trainers, pharmacies, churches, prisons, schools and colleges, community distributors, youth centres, salons and barber shops, drop-in centres, bars
South Africa	Pharmacies, hospitals, VCT sites, supermarkets (direct off the shelf), drop-in centres, CBOs, NGOs, private sector, community distributors, restrooms of bars, offices and other organizations, market in the same way as condoms

Respondents from all three countries supported distribution of self-tests through health facilities. Malawi was strongly in favour of this approach. Nearly all Malawian respondents suggested that public health facilities or existing non-governmental organizations (NGOs) and Community-based organizations (CBOs) working in HIV testing were the preferred distribution point for self-test kits.

Across all three settings, the advantages for facility-based distribution included possibilities for pre-and post-test counselling; greater potential for tracking test distribution, easier linkage to care and appropriate storage of test kits. Limitations of using health facilities were that healthcare workers are already overburdened, too busy to do counselling, and have a reputation for being judgmental. The other counter-argument against facility distribution was that it undermined the true value of self-testing: the tendency of many target groups of the HIVST to avoid facilities and interaction with healthcare personnel.

Respondents from all countries felt that community health workers who have previously had a role in HIV testing, could be potentially useful in a self-testing model, as they can explain how to perform the test, can offer counselling and advice on potential follow-up services, but need not know the result. As explained by this hypothetical example:

You have given me the kits, but you [the healthcare workers] don't know whether I have tested positive or negative which is very different from if I come to you to take the test. Because if I come to you to take the test you are the one who did the test and you know the result (Kenya; Procurement)

Pharmacies were also a popular potential distribution point for self-testing kits, especially by South African respondents. Pharmacies provided people the freedom to procure a self-test almost anywhere (in urban areas) at their convenience and without being recognized. Not being able to ensure counselling and linkage to care were frequently cited limitations for pharmacy and all other private distributors. Other innovative distribution approaches included supermarkets, in the restrooms of bars, offices and organizations or to co-market them with condoms. Overall, respondents felt that distribution of self-tests could be empowering by improving access to HIV testing and reducing the inconvenience associated with facility-based HTC.

Country-specific suggestions regarding distribution emerged. Kenya felt that the government would have to create an “enabling environment” for distribution of HIVST in the private sector. Respondents in Malawi anticipated that the procurement and distribution of self-tests were likely to be a major problem given regular stock-outs of existing HIV tests in the country. This concern was not raised by South African or Kenyan respondents.

#### Cost

In all settings there was a strong feeling that the government should be responsible for procurement of the tests and setting the payment mechanism. Usually this meant that at least some tests should be provided free to the user. Respondents did not generally think that it would be feasible for the government to bear the full costs of free distribution of self-tests in any setting (see Table 6).

**Table 6:** Quotations relating to costs/co-funding of test kits

Kenya	“We don't want a high cost … So would I be willing to buy a kit instead of a loaf bread? That is what it will come down to. If that is the choice the question is what will someone choose, will they choose the test or a loaf of bread?” (Academic)
Malawi	“In my opinion, if we are to achieve this, I think they should be for free just like the other models of testing.” (Government) “I would think it would be good if the user is able to pay for it because already I feel tax payers money is overburdened … but maybe we can subsidise the cost of the kit so that its affordable” (Government)
South Africa	“Government would be willing to pay for it if there is evidence that it really is increasing access, donors would also have a role” (Government) “People will happily pay R50 (approximately $5) not to have to go and sit in a queue all day to get their HIV status “(Academic)

Kenyan and South African respondents drew parallels between the distribution of self-tests and condoms. While condoms are freely available, some groups prefer to purchase them from pharmacies and other vendors for reasons of privacy or convenience. Respondents felt that some people would be willing to pay for the tests.

### Concerns, challenges and perceived evidence gaps

Despite the enthusiasm for self-testing, respondents from all countries suggested that further evidence and piloting was required. In addition, consultation with a broad range of stakeholders such as traditional leaders, health workers, academics, government, NGOs, donors and volunteers was considered an important next step in scale-up of the approach.

Some respondents raised concerns that large numbers of people, newly identified HIV-positive through HIVST could cause unanticipated demand for HIV services; thus further preparation of and investment into overburdened health services was required for self-testing to work.

#### Linkage to counselling and care

Perceptions on linkage focused on counselling in all three contexts, rather than on the uptake of care and treatment after HIVST (see Table 7). Counselling was deemed essential for first-time testers, who may constitute a sizeable proportion of HIVST users, particularly in the first few years after introduction. All three countries raised concerns about counselling: including the risk of suicide without it and ensuring it is of good quality. A few respondents, especially in South Africa, thought that the introduction of self-testing was not viable at all unless these counselling considerations were ensured.

I can only see HIV tests with proper good pre- and post-counselling and that has to be guaranteed (South Africa; NGO)

Suggested approaches to replace standard face-to-face counselling included a toll-free telephone helpline; test distributors and community health workers to offer information when distributing tests and group pre-test counselling sessions.

**Table 7:** Quotations relating to linkage to counselling and care

Kenya	“by the time we introduce it we want all that information very clearly out in the public that if you are alone and doing this test and you are worried you have this fears there is always this number you can call and you know get the help, you are not alone in this.” (Government) “That is where the problem is, getting this people to understand that after the test there is care and treatment and support. That is why I was insisting on that (helpline) number being on the kit.” (NGO)
Malawi	“what would be critical is that anyone who wants to access the self-testing kit should first undergo counselling so that whatever the results they should have at least undergone that process of being able to understand the test they are about to take.” (Government)
South Africa	“… there needs to be clear instructions of how to get into care, what needs to be done if you test positive and if there could be a reliable helpline to call, that would be ideal. At the moment, there isn't a reliable helpline to call” (NGO) “… with HIV self-test there should be intensified publicity, education, awareness that you can test yourself. This is what you will do, this is where you will go to get your treatment or where you can get advice.” (Academic)

Respondents felt that clear information and instructions must accompany all self-tests, but there was a gap in the evidence around what this should look like. They felt guidance should contain information about confirmatory testing; explain the window period; the risk of false negatives and false positives and how to link to care and treatment. Yet for all three countries, the inclusion of comprehensive and comprehensible information on the product insert was likely to be challenging given the number of languages spoken, literacy levels and the complexity of the messaging required in interpreting these screening tests.

#### Human rights and risk of coercion

As illustrated in Table 8, South African and Kenyan respondents more than those from Malawi voiced substantial concerns regarding human rights violations or abuse that may occur as a result of self-testing predicting that people would be forced to test at home [for example, wives, domestic servants, children, potential abuse victims], or the workplace (potential employees). It was recognized that coercion was not unique to self-testing, but the risk was greater with HIVST than other testing models. In Malawi, there was less probing on the issue of human rights abuses.

**Table 8:** Quotations relating to potential abuses

Kenya	“I say [there will be] violence. It will be forced, there will be people who will test others by force” (Procurement) “You need to do a lot of community awareness, because we know testing is not supposed to be mandatory.” (Government)
Malawi	[Not a major concern]
South Africa	“… there will be forced testing, but it is worth these risks, which already exist.” (Academic) “I can't see any legal reason to stop HIV self-testing. I can see lots of reluctance on the part of human rights people, as well as on the part of government. It's more the human rights people, an instinct around abuse. It's always about protecting the tiny percentage of people who are going to be abused.” (Academic)

While respondents from the three countries emphasized the potential for harm in varying degrees, all felt that self-testing could be empowering, giving people control over decision making, over their bodies and over their lives (see Table 9).

**Table 9:** Quotations relating to empowerment

Kenya	“When it comes to autonomy, it's even better. You choose the time, the place. You choose the scenario for yourself” (NGO)
Malawi	“… I see (HIVST) increasing couples’ talking, and in a way we would actually probably reduce GBV (gender-based violence) because sometimes that's the problem. When one goes for a test and the other doesn't know and then the other one does find out, it is always detrimental” (NGO)
South Africa	“Some [clients] have said it is a good idea because it empowers (them) to own their body and to take responsibility” (NGO)

## Discussion

Our findings indicate that stakeholders in Kenya, Malawi and South Africa showed considerable interest in introducing HIVST as a method to increase access to testing for hard-to-reach, high-risk populations. Policy makers supported HIVST in principal and there were surprising similarities across contexts. This was accompanied by several caveats and a need for additional contextual evidence on some issues. Respondents from Malawi, reflecting on current experience with HIVST at the community level, had a greater degree of apprehension, predicting hurdles before the scale-up of self-testing in their country. Respondents from Kenya and South African expressed more readiness but raised challenges over regulatory and policy issues. Concerns over potential social harms were expressed by respondents from all countries but issues around domestic violence and abuse where mentioned more frequently in the South African context.

### HIVST: reaching the untested

As others have shown, study stakeholders believed that self-testing could be an empowering strategy for reaching users concerned with stigma, confidentiality and the inconvenience of facility-based HTC [2,20,22,34]. Stakeholders felt that hard-to-reach groups such as men, MSM, couples and young people, who typically don't access facility-based HTC, would be the ideal beneficiaries of a self-testing programme. While community HTC approaches have attempted to address this need and show good rates of uptake, including among couples, the positivity rates are low and the data on linkage to care (especially among those who are negative) and on uptake among key populations are extremely limited [12,35,36]. Community approaches have been associated with improved social norms and reduction of stigma around testing [37], which our stakeholders identified as a key determinant in the desire for HIVST. Our data further highlights the need to better understand the ideal self-tester and their motivations.

### The effect of HIVST on linkage is unclear

In contrast to the belief that HIVST can expand testing of target populations through the freedom and opportunity to test at one's convenience, in any location [22,31,38,39] key informant stakeholders in all contexts strongly advocated the health facility route. This may have been informed by concerns regarding the lack of counselling and poor linkage to care associated with self-testing [31,40]. The loss of this link has an impact on the possibilities for public health impact [2,30,41]. Low rates of linkage are already a concern in HTC [16–18] and this is heightened in the self-testing model where link between tester and health professional is no longer clear-cut. Promising early studies show that home initiation of Antiretroviral treatment (ART) can lead to very high rates of linkage to care after HIVST, but this is based on a semi-supervised model and significant additional input that may not be replicable to scale or if testing is unsupervised [21]. Our data therefore point towards HIVST country readiness predicated on a supervised approach, where testing and counselling remains under some control of the health care worker [31,40]. HIVST requires greater individual proactivity and independence than is currently required from facility and community-based approaches to ensure the user proceeds to post-test counselling and linkage to care [31]. HIVST users may have a mismatch with this view and may specifically choose HIVST to avoid the need for intrusive or repetitive counselling that does not meet their needs and may decide to enrol for care and treatment at a time when the personal benefit appears to outweigh the perceived risks of accessing services. A better understanding of user perspectives on the counselling and linkage to care requirements in the self-testing model warrants further investigation.

### Rethinking the 5 Cs of HTC

The WHO guiding principles of HTC outline 5 Cs that must always accompany HTC: consent, confidentiality, counselling, correct results and linkage to care [42]. However, with the implied consent of individuals buying or collecting their own kits, the automatic confidentiality afforded by self-testing and the opportunity to choose whether or not to access counselling, support, treatment and care, the dynamics around the testing session necessarily shift. Country HIVST programmes provide an opportunity to look at novel ways for addressing and accumulating evidence on uptake of counselling and testing, linkage to care and potential social harms in the context of unsupervised HIVST [31,40]. International policy no longer emphasizes individualized, in-depth pre-test counselling advocating instead for newer, simplified HTC models [2]. HIVST could utilize this less restrictive policy space by developing creative solutions for the lack of formal or in-person counselling. Alternative solutions could include the provision of toll-free numbers for counselling and linkage to care [28,31,43], the delivery of testing instructions, messages, information and counselling via the internet or via a mobile phone application [23,25,31,40].

### Evidence-based policy is still required

Some important reservations remain for many stakeholders in our study, who advised proceeding with caution and not introducing self-testing at scale before considering evidence from pilot programmes. An often cited barrier to HIVST which emerged here and elsewhere is the concern that unsupervised testing could lead to human rights violations or abuse [24,30]. A recent review shows that little if any evidence of potential and unintended psychological and social harm when testing and counselling are decoupled, as in HIVST [43]. Whilst further evidence may counter these perceptions, the issue points to a longer-standing debate between safeguarding the rights and autonomy of individuals at risk or infected with HIV and promoting broader public health goals [23,24,44].

Stakeholders also felt that policy and guideline development should consider regulation, marketing and distribution, ensure human rights, and critically, linkage to appropriate support, care and treatment [4,5,23]. Low literacy levels in some target groups, lack of appropriate regulatory systems or quality assurance guidelines for self-testing and distribution problems would also need context-specific consideration before scale up [4,5,31]. Issues around willingness to pay, social marketing and incentives are not yet clear-cut. As HIVST expands, the market is stimulated and HIVST becomes desirable these can be rigorously tested, the subject of a newly awarded multi-country research grant [45]. Cost also needs to be discussed in line with target groups economic status and consequent accessibility. As robust evidence emerges, normative WHO guidelines, national guidelines and regulatory frameworks are required to guide the process in those areas which are new or specific to self-testing.

### Limitations

We interviewed 54 participants from three different contexts and anticipated that we would get a lot of different points of view with regard to our topic. Overall, we were struck by the remarkable similarities between countries and the relative homogeneity between participants allowing us to reach data saturation more rapidly [45]. In addition, whilst we interviewed a range of respondents from six different sectors, the small number of respondents per category made comparisons between key informant groups within one country difficult. Stakeholders approached as key informants declined being interviewed, and donors in Kenya, the procurement agency in Malawi and laboratory staff in South Africa are therefore not represented. We took a number of steps to ensure trustworthiness, conducting an additional layer of analysis across contexts and both strong similarities and the importance of contextual understanding emerged. Accompanying research with lay users focused on usability and acceptability of HIVST prototypes [20]. There was also exploration of the desire and need for counselling, but the depth and detail of the lay user interviews do not address other gaps identified here around potential harms, desire for confidentiality and the impact of autonomy and responsibility on linkage.

## Conclusions

Given the increasingly positive global policy environment on HIVST, we set out to understand national-level preparedness in HIV endemic settings. Stakeholders in three countries in sub-Saharan Africa with different HIV prevalence rates and untested populations felt that HIVST will be an important complement to existing community and facility-based testing approaches if accompanied by the same essential components of any HIV testing service, including easy access to accurate information and linkages to care. There are many challenges in implementation still to be addressed within national contexts and the need for further research to provide the contextual and operational evidence to address the concerns and contribute to normative WHO guidance in future.

## Supplementary Material

What are the constraints and opportunities for HIVST scale-up in Africa? Evidence from Kenya, Malawi and South Africa
