# Supplementary material for: What are the constraints and opportunities for HIVST scale-up in Africa? Evidence from Kenya, Malawi and South Africa
Source: J Int AIDS Soc. 2015 Mar 20;18(1):19445. doi: 10.7448/IAS.18.1.19445 (PMC4369555; doi:10.7448/IAS.18.1.19445)
Supplement: What are the constraints and opportunities for HIVST scale-up in Africa? Evidence from Kenya, Malawi and South Africa [file JIAS-18-19445-s001.pdf]

Evaluation of operational characteristics of late stage prototypes of HIV rapid tests
Interview Guide for Key Stakeholders

Definition key stakeholder: government officials involved in HIV control/MOH, NGOs, advocacy groups and social marketing groups, donors, national and international level procurement agencies, academic researchers, hospital directors.

OBTAIN INFORMED CONSENT

TOPICS FOR DISCUSSION

General information

1. What is your professional designation?

2. What is your role in HIV control and/or management?

3. How long have you been working in HIV control and/or management?

Population and Access

A. Stakeholder Group: Policymaker, MOH

1. Do you think your government/ministry will be interested in introducing an HIV rapid diagnostic test designed for self-testing**? If yes, answer 1b. If no, skip 1b and answer 1c.**

   1b. If yes, why do you think your government/ministry will be interested in introducing an HIV rapid diagnostic test designed for self-testing**? (Skip 1c)**

   1c. If no, why do you think your government/ministry will not be interested in introducing an HIV rapid diagnostic test designed for self-testing**?**

   PROBE:
   Why or why not do you think a country should scale-up HIV self-testing?
   How ready do you think this country is for national, regional or district scale-up of HIV self-testing?
   What steps are needed before scale-up of HIV self-testing?

   Is there a need for HIV self-testing when there are other existing models of HIV testing that are under-utilized or poorly resourced? What would be reasons for using a new strategy?

2. What would be the most appropriate ways of distributing such an HIV self-test?
   What do you think would be best setting: Community or facility?

3. How do you think people would access an HIV self-testing kit?

CONFIDENTIAL

4. Are there specific population/groups that you believe would most likely use an HIV self-test in your country? If so, please name the population/groups most likely to use an HIV self-testing test/device in your country?

5. How would the government access these population/groups for HIV self-testing?

6. We are interested in inviting people from these population/groups to try some HIV self-test prototypes. How do you think we could access these population/groups?

7. Who do you think should pay for these HIV self-test kits device in your country?
   7.1 Would the government be willing to pay? If so, what would you estimate to be an acceptable price for the HIV self-test?
   7.2 Is direct user procurement reasonable?

8. What benefits do you see to the introduction of an HIV self-test?
   PROBE:
   What do you think would influence the adoption of HIV self-testing?
   Would WHO guidance and frameworks on HIV testing be helpful?

9. What challenges do you foresee with the introduction of an HIV self-test?
   PROBE:
   Do you think there are potential harms? If yes, do you think the potential risks outweigh the benefits of introducing HIV self-tests?
   Which different interest groups should be included in this debate on the challenges and benefits of introducing HIV self-tests?
   How do you think laboratory and other professional groups will react to the introduction of HIV self-tests?

   What do you think consent means in the context of HIV self-testing? What about autonomy?

   What are your suggestions on the dissemination of the results of this study especially usability of HIV self-test features/characteristics? How should the results be reported/disseminated so they can be used in decision making process on HIV self-testing in this country?

B. Stakeholder Group: Procurement/Distribution

1. How interested would your group be in an HIV rapid diagnostic test that is designed to enable HIV self-testing?

   Please explain why/why not?
   PROBE:
   Why or why not do you think a country should scale-up HIV self-testing?
   How ready do you think this country is for national, regional or district scale-up of HIV self-testing?
   What steps are needed before scale-up of HIV self-testing?

   Is there a need for self-testing when there are other existing models of HIV self-testing that are under-utilized or poorly resourced? What would be reasons for using a new strategy?

CONFIDENTIAL

2. Who do you think would be the typical user/beneficiary of an HIV self-test? Are there specific groups who would benefit most? If so, who?

3. What would be the most appropriate ways of distributing such an HIV self-test? What would be best setting: Community or facility?

4. How do you think people would access an HIV self-testing kit?

5. What do you think is the best way to access these population/groups for HIV self-testing?

6. We are interested in inviting people from these population/groups to try out some HIV self-test prototypes. How do you think we could access these population/groups?

7. Who do you suggest should pay for these HIV self-test kits in your country?
   7.1 Is direct user procurement reasonable?

8. What benefits do you see to the introduction of an HIV self-test?
   PROBE:
   What do you think would influence the adoption of HIV self-testing?
   Would WHO guidance and frameworks on HIV testing be helpful?

9. What challenges do you foresee with the introduction of an HIV self-test?
   PROBE:
   What do you think will be reaction of lab and regulatory frameworks?

C. Stakeholder Group: Advocate for Community and vulnerable patient group

1. How interested would your beneficiary population/groups be in an HIV rapid diagnostic test that is designed to enable HIV self-testing?

   Please explain why/why not?
   PROBE:
   Why or why not do you think a country should scale-up HIV self-testing?
   How ready do you think this country is for national, regional or district scale-up of HIV self-testing?
   What steps are needed before scale-up of HIV self-testing?

   Is there a need for HIV self-testing when there are other existing models of HIV testing that are under-utilized or poorly resourced?

   What would be reasons for using a new strategy?

2. Would an HIV self-test improve access to people who are under-diagnosed for social stigma reasons? Please provide reasons for your response.

3. Are there specific population/groups that you believe would be most likely to use an HIV self-test in your country? If so, please define the population/groups most likely to use an HIV self-testing test/device in your country?

CONFIDENTIAL

4. What would be the most appropriate ways of distributing these HIV self-test kits?
   What would be best setting: Community or facility?

5. How do you think people would access an HIV self-test?

6. What do you think is the best way to access these population/groups for HIV self-testing?
7. We are interested in inviting people from these population/groups to try out some HIV self-test prototypes. How do you think we could access these population/groups?

8. Who do you suggest should pay for these HIV self-test kits in your country?
   8.1. Is direct user procurement reasonable?

9. What benefits to do you see to the introduction of an HIV self-test?
   PROBE:
   What do you think would influence the adoption of HIV self-testing?
   Would WHO guidance and frameworks on HIV self-testing be helpful?

7. What challenges do you foresee with the introduction of an HIV self-test?
   PROBE:
   Which different interest groups should be included in the debate on the challenges and benefits of introducing HIV self-tests?

   How do you think laboratory and other professional groups will react to the introduction of HIV self-tests?

   What do you think consent means in the context of self-testing? What about autonomy?

   What are your suggestions on the dissemination of the results of this study especially usability of HIV self-test features/characteristics? How should the results be reported/disseminated so they can be used in decision making process on HIV self-testing in this country?

D. Stakeholder Group: Academic Researcher

1. Are there specific population/groups that you believe would be most likely to use an HIV self-test in your country? If so, please define the population/groups most likely to use an HIV self-testing test/device in your country?

   PROBE:
   Why or why not do you think a country should scale-up HIV self-testing?
   How ready do you think this country is for national, regional or district scale-up of HIV self-testing?
   What steps are needed before scale-up of HIV self-testing?

   Is there a need for self-testing when there are other existing models of HIV self-testing that are under-utilized or poorly resourced?

   What would be reasons for using a new strategy?

CONFIDENTIAL

2. What would be the most appropriate ways of distributing the HIV self-test kits?
   What would be best setting: Community or facility?

3. How do you think people would access an HIV self-test?

4. What do you think is the best way to access these population/groups for HIV self-testing?

5. We are interested in inviting people from these population/groups to try out some HIV self-test prototypes. How do you think we could access these population/groups?

6. Who do you suggest should pay for these HIV self-test kits in your country?
   - Is direct user procurement reasonable?

7. What benefits do you foresee with the introduction of an HIV self-test?
   PROBE:
   What do you think would influence the adoption of HIV self-testing?
   Would WHO guidance and frameworks on HIV self-testing be helpful?

8. What challenges do you foresee with the introduction of an HIV self-test?
   PROBE:
   Which different interest groups should be included in the debate on the challenges of introducing HIV self-tests?
   How do you think laboratory and other professional groups will react to the introduction of HIV self-tests?

   What do you think consent means in the context of HIV self-testing? What about autonomy?

   What are your suggestions on the dissemination of the results of this study especially usability of test features/characteristics? How should the results be reported/disseminated so they can be used in decisions about HIV self-testing in this country?

E. Stakeholder Group: Hospital directors/Health care provider (?)

1. How interested are you in an HIV rapid diagnostic test that is designed to enable HIV self-testing? Please explain why/why not?
   PROBE:
   Why or why not do you think a country should scale-up HIV self-testing?
   How ready do you think this country is for national, regional or district scale-up of HIV self-testing?
   What steps are needed before scale-up of HIV self-testing?

   Is there a need for HIV self-testing when there are other existing models of HIV testing that are under-utilized or poorly resourced?
   What would be reasons for using a new strategy?

CONFIDENTIAL

2. How would you recommend that patients interact with the health care system in the event of an HIV positive self-test result?

3. In your view, is there a “typical” patient for an HIV self-test? If so, how would you describe that patient?

4. What would be the most appropriate ways of distributing HIV self-test kits?
   What would be best setting: Community or facility?

5. How do you think people would access an HIV self-test?

6. We are interested in inviting people from these population/groups to try out some HIV self-test prototypes. How do you think we could access these population/groups?

7. How best can we access these population/groups for HIV self-testing? Or for the usability research?

8. Who do you think should pay for an HIV self-test in your country? Is direct user procurement reasonable?

9. What challenges do you foresee with the introduction of an HIV self-test?

   PROBE:
   Which different interest groups should be included in the debate on the challenges of introducing HIV self-tests?
   How do you think laboratory and other professional groups will react to the introduction of HIV self-tests?

   What do you think consent means in the context of HIV self-testing? What about autonomy?

   What are your suggestions on the dissemination of the results of this study especially usability of test features/characteristics? How should the results be reported/disseminated so they can be used in decisions about HIV self-testing in this country?

QUESTION FOR ALL STAKEHOLDERS:
Test characteristics

1. What do you think are most challenging technical/operational aspects of conducting an HIV self-test?

2. In your opinion, what characteristics of a HIV test are most important for selecting a test for HIV self-testing?

Probe on the following:

- Test performance e.g., sensitivity, specificity, detection of specific antibodies (HIV-1, HIV-2)
- Cost
- Acceptability – please specify what? Size e.g., discreet so fits into pocket?
- Sample – blood or oral/saliva
- Number of steps, integrated steps
- Instructions for using – pictorial, written

CONFIDENTIAL

- Time to results
- Ease of use, safety, need for training, shelf life
- Other…

Linkage to care

1. Effective linkage to care is often cited as a barrier to HIV self-testing. How do you think linkage to care could best be achieved with HIV self-testing?

Referrals

1. Can you please recommend two additional contacts that you feel we should interview for this topic? Name organization, contact information?

CONFIDENTIAL
